# CAF-secreted CXCL1 conferred radioresistance by regulating DNA damage response in a ROS-dependent manner in esophageal squamous cell carcinoma

**DOI:** 10.1038/cddis.2017.180

**Published:** 2017-05-18

**Authors:** Hongfang Zhang, Jing Yue, Zhenzhen Jiang, Rongjing Zhou, Ruifei Xie, Yiping Xu, Shixiu Wu

**Affiliations:** 1 Hangzhou Cancer Institution, Hangzhou Cancer Hospital, Hangzhou 310002, China; 2 Department of Pathology, Hangzhou Cancer Hospital, Hangzhou 310002, China; 3 Department of Bio-informatics, Hangzhou Cancer Hospital, Hangzhou 310002, China

## Abstract

Five-year survival rate of esophageal squamous cell carcinoma (ESCC) patients treated with radiotherapy is <20%. Our study aimed to investigate whether cancer-associated fibroblasts (CAFs), one major component of tumor microenvironment, were involved in tumor radioresistance in ESCC. By use of human chemokine/cytokine array, human chemokine CXCL1 was found to be highly expressed in CAFs compared with that in matched normal fibroblasts. Inhibition of CXCL1 expression in CAFs significantly reversed CAF-conferred radioresistance *in vitro* and *in vivo*. CAF-secreted CXCL1 inhibited the expression of reactive oxygen species (ROS)-scavenging enzyme superoxide dismutase 1, leading to increased ROS accumulation following radiation, by which DNA damage repair was enhanced and the radioresistance was mediated. CAF-secreted CXCL1 mediated the radioresistance also by activation of Mek/Erk pathway. The cross talk of CAFs and ESCC cells induced CXCL1 expression in an autocrine/paracrine signaling loop, which further enhanced tumor radioresistance. Together, our study highlighted CAF-secreted CXCL1 as an attractive target to reverse tumor radioresistance and can be used as an independent prognostic factor of ESCC patients treated with chemoradiotherapy.

Esophageal carcinoma is an aggressive cancer with the eighth incidence and sixth mortality in the world.^1, 2^ There are two histological subtypes of esophageal carcinoma, including esophageal squamous cell carcinoma (ESCC) and esophageal adenocarcinoma. In Asian countries, ESCC accounted for 79% of total ESCC cases worldwide.^3^ As early symptoms are not conspicuous, more than 70% of ESCC patients were diagnosed at middle or late stage. For these patients, radiotherapy is used as one major curative treatment modality. However, 5-year survival rate of ESCC patients after radiotherapy is <20% as a result of tumor radioresistance.^4^ Previous studies have focused on tumor cells to discover molecular targets to reverse radioresistance. However, the host tumor microenvironment (TME) has been completely ignored.^5, 6, 7, 8, 9, 10^ TME is composed of several types of non-tumor cells such as cancer-associated fibroblasts (CAFs), immune cells and endothelial cells.^11^ Accumulating evidences have suggested that TME is involved in tumor initiation and progression via the complicated cross talk with tumor cells. CAFs, as one major component of TME, have gained increasing attention for their important roles in determining tumor cells’ fate.^12, 13^ Whether CAFs affected tumor radioresponse has remained never to be studied in ESCC.

Chemokines expressed in tumor cells or stromal cells are a class of small-molecular chemotactic cytokines and regulate cell trafficking and positioning.^14^ Aberrant expressions of chemokines and their cognate receptors were associated with several human diseases such as cancer, autoimmune and inflammatory diseases. Furthermore, chemokines were found to have vital roles in cell–cell interaction by recruitment and activation of different cell types to tumor sites via autocrine or paracrine mechanisms.^15, 16^ The chemokines expressed in stromal cells were demonstrated to be implicated in tumor growth, metastasis and angiogenesis.^15^ In ESCC, several chemokines including CXCL12, CXCL8 and CXCL10 were discovered as potential tumor biomarkers.^17^ Some of these chemokines significantly correlated with tumor size, lymph node involvement and distant metastases of ESCC patients.

In our study, we investigated whether CAF-secreted chemokines were involved in tumor radioresponse and how chemokines were involved in the interaction of CAFs and tumor cells. By *in vitro* and *in vivo* studies, human chemokine CXCL1 was discovered as an attractive target to reverse CAF-conferred radioresistance. Further studies revealed that CAF-secreted CXCL1 conferred tumor radioresistance by activation of DNA damage repair and Mek/Erk signaling pathway. Moreover, CXCL1 expressed in CAFs was found to be an independent prognostic factor of ESCC patients treated with chemoradiotherapy.

## Results

### CXCL1 was highly expressed in CAFs in comparison with that in matched normal fibroblasts

**Figure 1:**
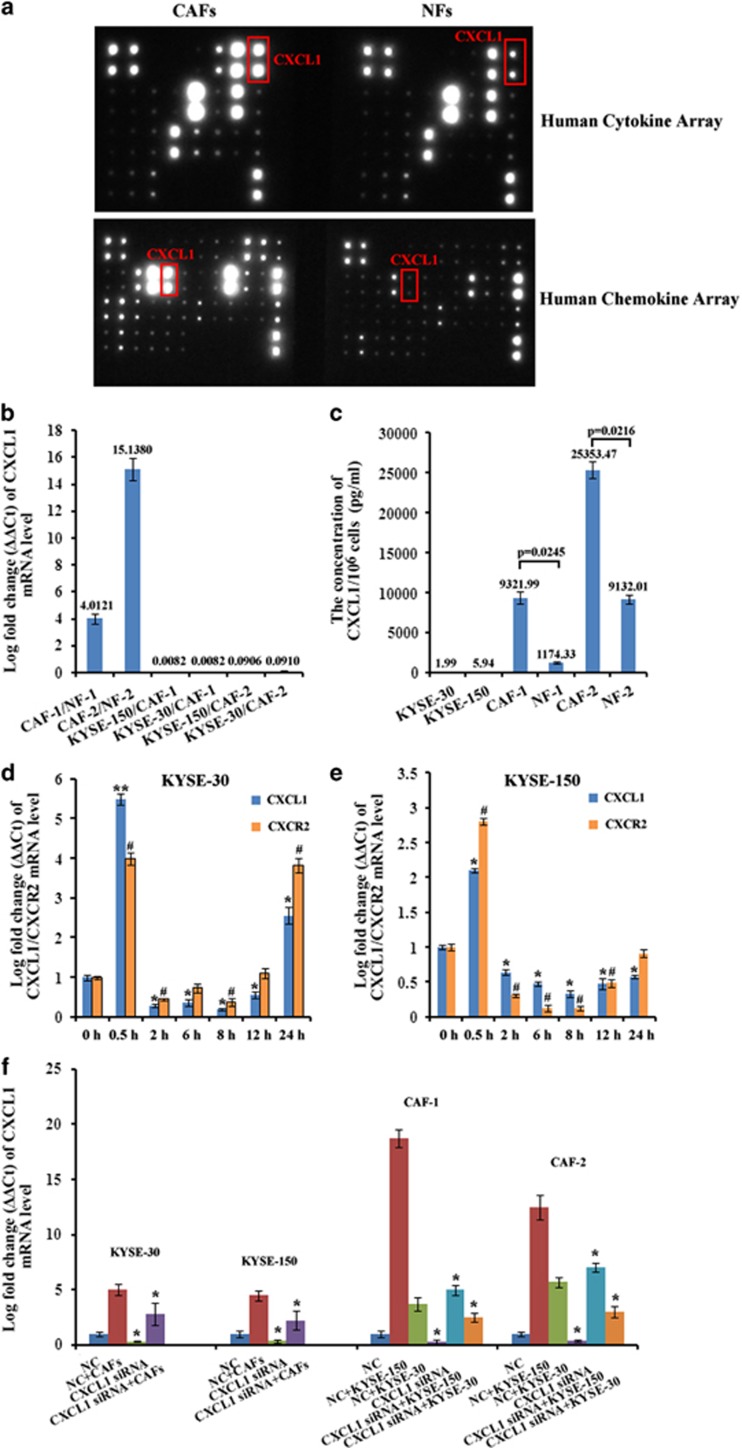
Human chemokine CXCL1 was expressed in an autocrine/paracrine signaling loop. (**a**) CXCL1 was highly expressed in CAF medium compared with in matched NF medium as determined with human cytokine/chemokine array. (**b**) The fold change of CXCL1 mRNA level in different cells by qRT-PCR analysis. (**c**) The concentration of CXCL1 in culture medium of KYSE-30, KYSE-150, CAF-1, NF-1, CAF-2 and NF-2 by ELISA. (**d** and **e**) The mRNA level changes of CXCL1 and its receptor CXCR2 after 8 Gy of radiation in KYSE-30 and in KYSE-150 by qRT-PCR analysis. *P<0.05, **P<0.01, compared with CXCL1 mRNA level in KYSE-30 or KYSE-150 before radiation. ^#^*P*<0.05, compared with CXCR2 mRNA level in KYSE-30 or KYSE-150 before radiation. (**f**) The fold change of CXCL1 mRNA level in KYSE-30 and KYSE-150 that were cultured in CAF medium for 24 h compared with cultured in normal medium, and in CAF-1 and in CAF-2 that were cultured in the CM of KYSE-30 or KYSE-150 for 24 h compared with cultured in normal medium by qRT-PCR analysis. Negative control (NC): the cells that were transfected with scrambled siRNA. *P<0.05, compared with CXCL1 mRNA level in NC that was transfected with scrambled siRNA before cultured in normal medium for 24 h

As the family of human chemokines was evidenced to have important roles in TME and in the cross talk of tumor cells and their host microenvironment.^18^ Our study investigated whether human chemokines were differentially expressed in CAFs and in matched normal fibroblasts (NFs; their characterization was shown in Supplementary Information). By use of human chemokine/cytokine array, we found CXCL1 was highly expressed in CAFs medium compared with in matched NFs (Figure 1a and Supplementary Figure S2). By quantitative RT-PCR (qRT-PCR) analysis, the mRNA level of CXCL1 showed an increase of 4.0121- and 15.1380-fold in CAF-1 and CAF-2 compared with that in NF-1 and NF-2, respectively (Figure 1b). However, CXCL1 was far less expressed in tumor cells KYSE-30 and KYSE-150 (Figure 1b). By enzyme-linked immunosorbent assay (ELISA), the concentration of CXCL1 was found to be significantly higher in CAF medium compared with that in matched NF medium (CAF-1: 9321.99 pg/ml; NF-1: 1174.33 pg/ml; *P*=0.0245; CAF-2: 25353.47 pg/ml; NF-2: 9132.01 pg/ml; *P*=0.0216), while extremely low in the culture medium of tumor cells (Figure 1c). These results suggested the major source of CXCL1 was CAFs but not tumor cells. Furthermore, we found the expressions of CXCL1 and its receptor CXCR2 were significantly increased 30 min after 8 Gy of radiation in KYSE-150 and KYSE-30, which may cause a constitutively activated CXCL1/CXCR2 signaling in tumor cells (Figures 1d and e).

When cultured in CAF medium for 24 h, the expression of CXCL1 in KYSE-30 and KYSE-150 showed an increase of 2.8102- and 2.2139-fold, respectively, compared with cultured in normal medium (Figure 1f); CXCL1 was also significantly upregulated in CAF-1 and CAF-2 that were cultured in the conditioned medium (CM) of tumor cells for 24 h, compared with cultured in normal medium (Figure 1f). These results suggested the cross talk of CAFs and tumor cells may result in CXCL1 expression in an autocrine/paracrine signaling loop.

### CXCL1 conferred radioresistance by enhancement of cellular DNA damage repair

**Figure 2:**
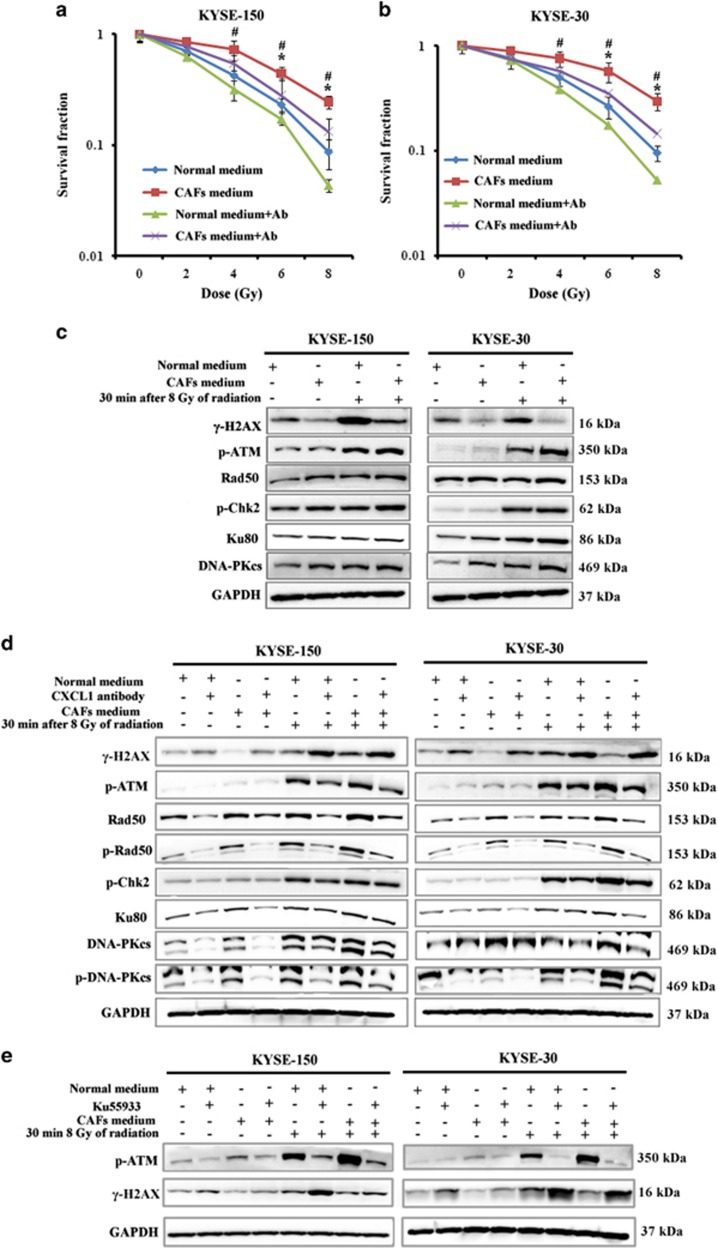
CXCL1 conferred radioresistance by enhancement of DNA damage repair. (**a** and **b**) The determination of the radiosensitivity of KYSE-150 and KYSE-30 that were cultured in normal medium or in CAF medium with or without 500 ng/ml CXCL1 antibody for 24 h by clonogenic survival assay. *P<0.05, compared with cultured in CAF medium with 500 ng/ml CXCL1 antibody. ^#^*P*<0.05, compared with cultured in normal medium. Ab, 500 ng/ml CXCL1 antibody. (**c**) The expressions of cellular DNA damage repair proteins including *γ*-H2AX, p-ATM, Rad50, p-Chk2 and DNA-PKcs 30 min after radiation in KYSE-150 and in KYSE-30 that were cultured in normal medium or in CAF medium for 24 h, by western blotting analysis. GAPDH was used as a loading control. (**d**) The expressions of *γ*-H2AX, p-ATM, Rad50, p-Rad50, p-Chk2, Ku80, DNA-PKcs and p-DNA-PKcs 30 min after radiation in KYSE-150 and in KYSE-30 that were cultured in normal medium or in CAF medium with or without 500 ng/ml CXCL1 antibody for 24 h, by western blotting analysis. GAPDH was used as a loading control. (**e**) The expressions of p-ATM and *γ*-H2AX 30 min after radiation in KYSE-150 and KYSE-30 that were cultured in normal medium or in CAF medium with or without 10 *μ*M ATM kinase inhibitor Ku55933 for 24 h, by western blotting analysis. GAPDH was used as a loading control

When cultured in CAF medium, both KYSE-30 and KYSE-150 displayed significant radioresistance compared with when cultured in normal medium (Figures 2a and b). When KYSE-30 and KYSE-150 were cultured in CAFs medium, which contained 500 ng/ml CXCL1 antibody, the radioresistance of tumor cells was significantly reversed, suggesting CAF-secreted CXCL1 was responsible for tumor radioresistance (Figures 2a and b).

Our study further explored how CAF-secreted CXCL1 conferred tumor radioresistance. We found the expression of *γ*-H2AX, a marker of DNA double-strand breaks, which are one major form of cellular damage induced by radiation, was decreased after 8 Gy of radiation in KYSE-30 and KYSE-150 that were cultured in CAF medium for 24 h; meanwhile, cellular DNA damage repair proteins including p-ATM, Rad50, p-Chk2, Ku80 and DNA-PKcs were upregulated after 8 Gy of radiation in KYSE-30 and KYSE-150 that were cultured in CAF medium for 24 h (Figure 2c and Supplementary Figure S3). Upon inhibition of CAF-secreted CXCL1 by 500 ng/ml CXCL1 antibody, the expression of *γ*-H2AX was increased while the expressions of cellular DNA damage repair proteins were decreased after 8 Gy of radiation in KYSE-30 and KYSE-150 that were cultured in CAF medium for 24 h (Figure 2d). These results suggested CAF-secreted CXCL1 enhanced DNA damage repair after radiation.

**Figure 3:**
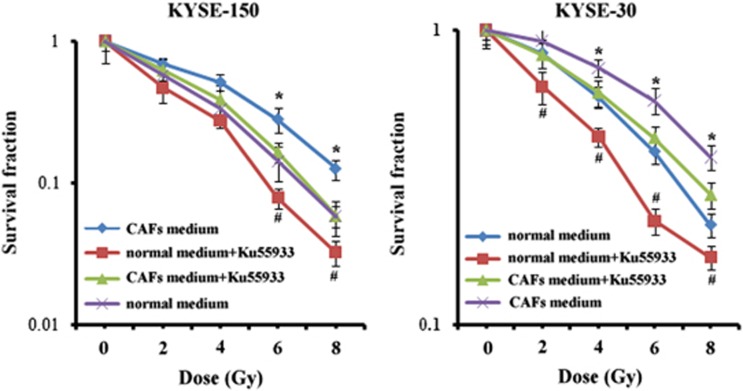
Inhibition of ATM kinase reversed CAF-conferred radioresistance. The radiosensitivity of KYSE-150 and KYSE-30 that were cultured in normal medium or in CAF medium with or without 10 *μ*M ATM kinase inhibitor Ku55933 for 24 h as determined by clonogenic survival assay. *P<0.05, compared with cultured in CAF medium with 10 *μ*M ATM kinase inhibitor Ku55933. ^#^*P*<0.05, compared with cultured in normal medium without 10 *μ*M ATM kinase inhibitor Ku55933

Our study investigated whether attenuation of DNA damage repair by 10 *μ*M ATM kinase inhibitor Ku55933 could reverse CXCL1-conferred tumor radioresistance. The expression of *γ*-H2AX was obviously increased after 8 Gy of radiation in KYSE-30 and KYSE-150 that were cultured in CAF medium with ATM inhibitor Ku55933 for 24 h (Figure 2e). Furthermore, treatment with 10 *μ*M ATM kinase inhibitor Ku55933 significantly decreased clonogenic survival of KYSE-150 and KYSE-30 that were cultured in CAF medium before radiation (Figure 3). These results suggested CAF-secreted CXCL1 conferred tumor radioresistance by enhancement of DNA damage repair.

### CXCL1 enhanced cellular DNA damage repair in a superoxide dismutase 1–reactive oxygen species-axis-dependent manner

**Figure 4:**
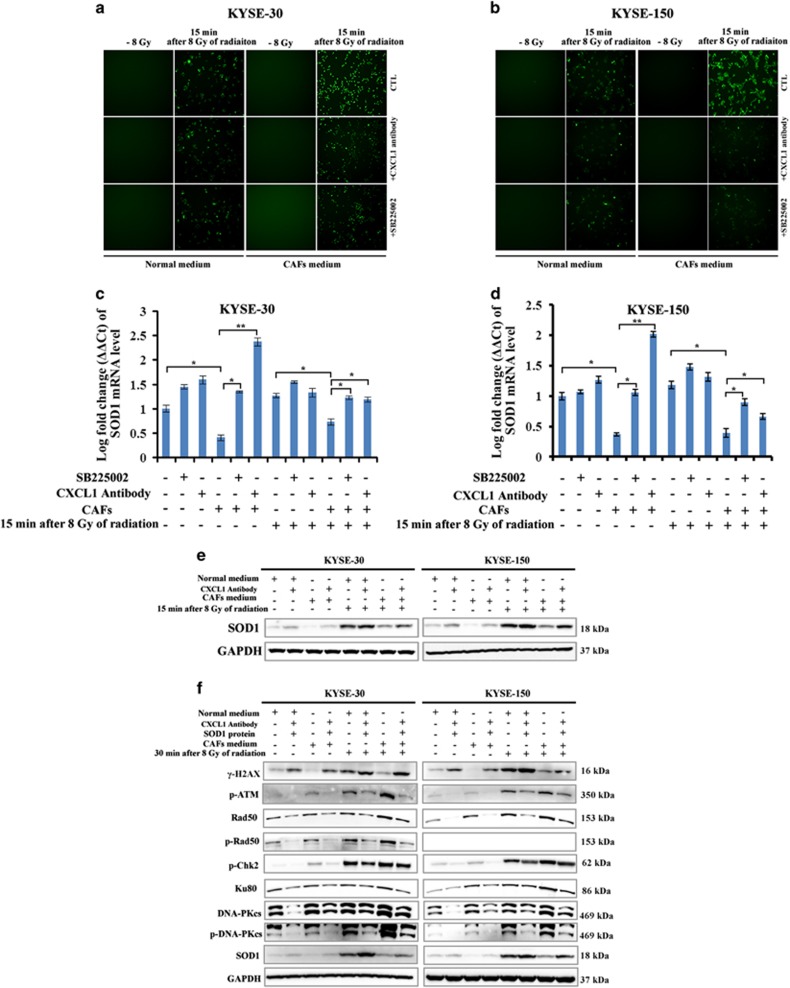
CXCL1 enhanced DNA damage repair in a SOD1–ROS-axis-dependent manner. (**a** and **b**) The ROS level following radiation in KYSE-150 and in KYSE-30 that were cultured in normal medium or CAF medium with or without 500 ng/ml CXCL1 antibody or 400 nM CXCR2 inhibitor SB225002 for 24 h, by immunofluorescence analysis. Magnification: × 10. (**c** and **d**) The fold change of SOD1 mRNA level before or after radiation in KYSE-150 and in KYSE-30 that were cultured in normal medium or in CAF medium with or without 500 ng/ml CXCL1 antibody or 400 nM CXCR2 inhibitor SB225002 for 24 h, by qRT-PCR analysis. *P<0.05, **P<0.01. (**e**) The expression of SOD1 protein following radiation in KYSE-150 and in KYSE-30 that were cultured in normal medium or in CAF medium with or without 500 ng/ml CXCL1 antibody for 24 h, by western blotting analysis. GAPDH was used as a loading control. (**f**) The expressions of *γ*-H2AX, p-ATM, Rad50, p-Rad50, p-Chk2, Ku80, DNA-PKcs, p-DNA-PKcs and SOD1 following radiation in KYSE-150 and in KYSE-30 that were cultured in normal medium or in CAF medium with or without 100 ng/ml human SOD1 protein for 24 h, by western blotting analysis. GAPDH was used as a loading control

When KYSE-30 and KYSE-150 were cultured in CAF medium, reactive oxygen species (ROS) was obviously increased 15 min after 8 Gy of radiation compared with when cultured in normal medium (Supplementary Figure S4). Upon blockage of CAF-secreted CXCL1 signaling by 500 ng/ml CXCL1 antibody or by 400 nM CXCR2 inhibitor SB225002, CAF-enhanced ROS increase following radiation was attenuated in KYSE-30 and in KYSE-150, suggesting CAF-secreted CXCL1 enhanced ROS increase following radiation in tumor cells (Figures 4a and b). As ROS could initiate the activation of cellular DNA damage repair,^19^ our study investigated whether CAF-secreted CXCL1 enhanced DNA damage repair by increasing ROS. Several molecular and signaling pathways are responsible for cellular ROS balance, among which superoxide dismutases (SODs) are one of the chief ROS-scavenging enzymes.^20^ We found the mRNA level of SOD1 in KYSE-30 and KYSE-150 was significantly decreased either before or after radiation when cultured in CAF medium (Figures 4c–e). When 500 ng/ml CXCL1 antibody or 400 nM CXCR2 inhibitor SB225002 was added into CAF medium, SOD1 was significantly upregulated in tumor cells (Figures 4c–e), suggesting CAF-secreted CXCL1 inhibited the expression of SOD1. When cultured in CAF medium, which contained 100 ng/ml SOD1 protein, the increase of ROS following radiation was obviously attenuated in KYSE-30 and in KYSE-150, suggesting CAF-secreted CXCL1 enhanced radiation-induced ROS increase in a SOD1-dependent manner (Supplementary Figure S5). Furthermore, we found the expression of *γ*-H2AX was increased while the expressions of cellular DNA damage repair proteins were decreased following radiation when KYSE-30 and KYSE-150 were cultured in CAFs medium, which contained 100 ng/ml SOD1 protein (Figure 4f). These results suggested CAF-secreted CXCL1 enhanced DNA damage repair in a SOD1–ROS-axis-dependent manner.

### CXCL1 conferred radioresistance by activation of Mek/Erk signaling pathway

**Figure 5:**
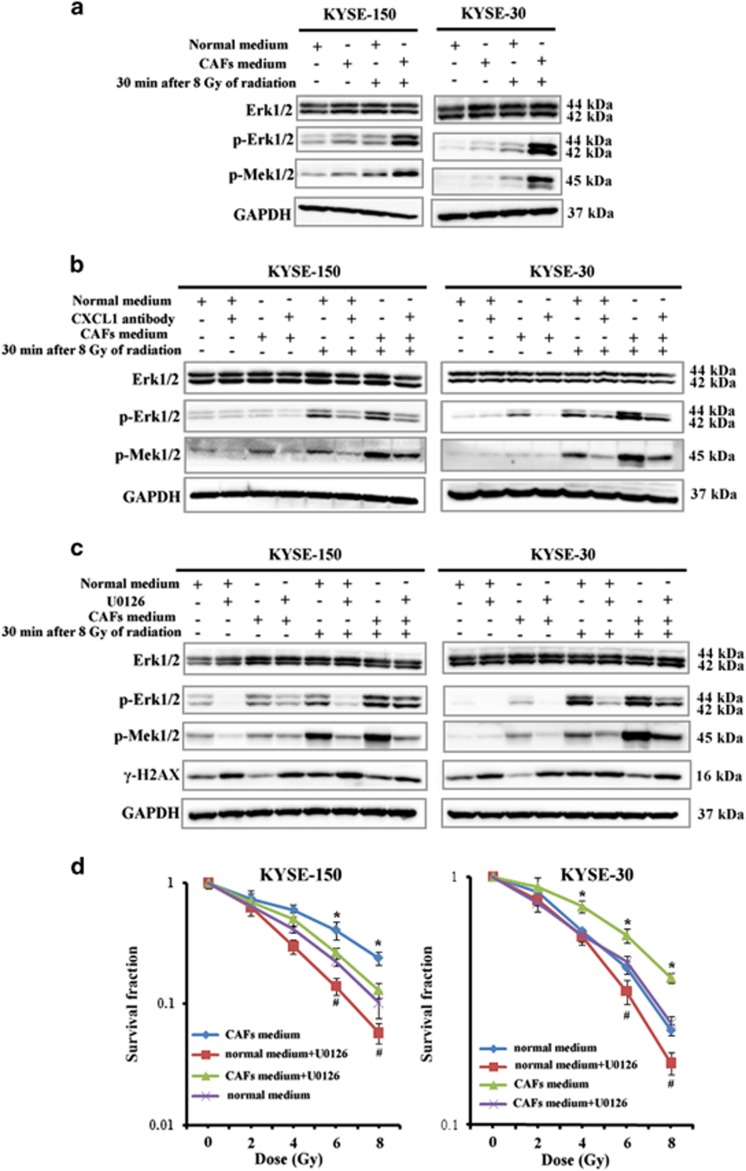
CXCL1 conferred radioresistance by activation of Mek/Erk signaling pathway. (**a**) The expressions of Erk1/2, p-Erk1/2 and p-Mek1/2 following radiation in KYSE-150 and in KYSE-30 that were cultured in normal medium or in CAF medium for 24 h, by western blotting analysis. GAPDH was used as a loading control. (**b**) The expressions of Erk1/2, p-Erk1/2 and p-Mek1/2 following radiation in KYSE-150 and in KYSE-30 that were cultured in normal medium or in CAF medium with or without 500 ng/ml CXCL1 antibody for 24 h, by western blotting analysis. GAPDH was used as a loading control. (**c**) The expressions of Erk1/2, p-Erk1/2, p-Mek1/2 and *γ*-H2AX following radiation in KYSE-150 and in KYSE-30 that were cultured in normal medium or in CAF medium with or without 10 *μ*M Mek1/2 kinase inhibitor U0126 for 24 h, by western blotting analysis. GAPDH was used as a loading control. (**d**) The radiosensitivity of KYSE-150 and KYSE-30 that were cultured in normal medium or in CAF medium with or without 10 *μ*M Mek1/2 kinase inhibitor U0126 for 24 h as determined by clonogenic survival assay. *P<0.05, compared with cultured in CAF medium with 10 *μ*M Mek1/2 kinase inhibitor U0126. ^#^*P*<0.05, compared with cultured in normal medium without 10 *μ*M Mek1/2 kinase inhibitor U0126

When KYSE-30 and KYSE-150 were cultured in CAF medium, the activation of Erk1/2 following radiation was increased as a result of increased phosphorylation of Mek1/2, suggesting CAFs activated Mek/Erk signaling pathway in tumor cells (Figure 5a). Total Erk1/2 expression was not obviously changed in KYSE-30 and in KYSE-150 when cultured in CAF medium (Figure 5a). When KYSE-30 and KYSE-150 were cultured in CAF medium, which contained 500 ng/ml CXCL1 antibody, the activation of Mek/Erk signaling pathway following radiation were repressed, suggesting CAF-secreted CXCL1 could activate Mek/Erk signaling pathway (Figure 5b). Previous studies suggested Mek/Erk signaling pathway was involved in tumor radioresponse.^21^ Herein, out study investigated whether CXCL1-activated Mek/Erk signaling pathway was involved in tumor radioresistance. When KYSE-30 and KYSE-150 were cultured in CAF medium, which contained 10 *μ*M Mek1/2 kinase inhibitor U0126, the expression of *γ*-H2AX after radiation was increased following the inhibition of Mek/Erk signaling pathway (Figure 5c). Furthermore, treatment with 10 *μ*M Mek1/2 kinase inhibitor U0126 significantly decreased clonogenic survival of KYSE-150 and KYSE-30 that were cultured in CAF medium before radiation ((Figure 5d). These results suggested CAF-secreted CXCL1 conferred tumor radioresistance also by activation of Mek/Erk signaling pathway.

### Inhibition of CAF-secreted CXCL1 reversed radioresistance of xenograft tumor models

**Figure 6:**
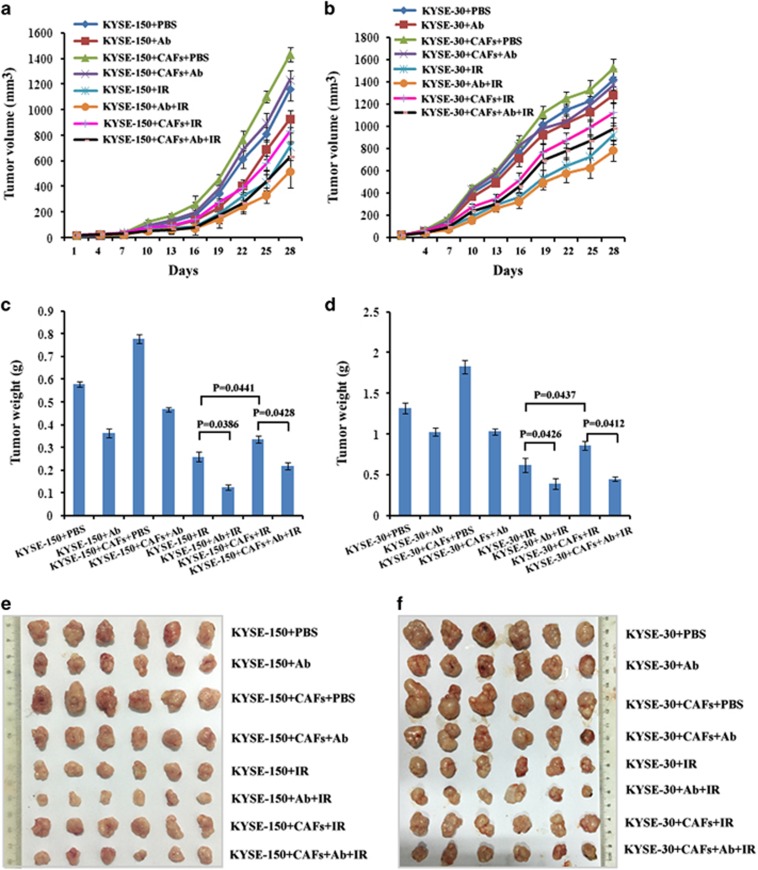
Blockage of CAF-secreted CXCL1 reversed radioresistance of xenograft tumor models. (**a** and **b**) The growth curve of xenograft tumors implanted with KYSE-150 or KYSE-30 alone or combined with CAFs after treatment with tumor injection of 1 *μ*g/ml CXCL1 antibody, fractionated radiation at a total dose of 12 Gy alone or their combinations. Tumors treated with only PBS was chosen as a control. (**c** and **d**) The weight of excised xenograft tumors in (**a**) and (**b**) at the end of experiment at day 28. (**e** and **f**) The photos of excised xenograft tumors in (**a**) and (**b**) at the end of experiment at day 28

In order to investigate CAF-secreted CXCL1 conferred radioresistance *in vivo*, xenograft tumor models implanted with tumor cells alone or in combination with CAFs had been established in BALB/c nude mice. We found the volume and weight of xenograft tumors after radiotherapy were larger in groups that were implanted with tumor cells combined with CAFs compared with in groups that were implanted with tumor cells alone (Figure 6). These results suggested CAFs conferred tumor radioresistance *in vivo*. Our study further investigated whether blockage of CAF-secreted CXCL1 could reverse *in vivo* radioresistance. When treated with the combination therapy with radiotherapy and CXCL1 antibody, the volume and weight of xenograft tumors were both decreased in comparison with radiotherapy alone (Figure 6). The inhibition rate (IR) of radiotherapy was significantly increased when combined with CXCL1 antibody (Supplementary Figure S6). These results suggested inhibition of CAF-secreted CXCL1 reversed tumor radioresistance *in vivo*.

### CXCL1 was an independent prognostic factor of ESCC patients treated with radiotherapy

**Figure 7:**
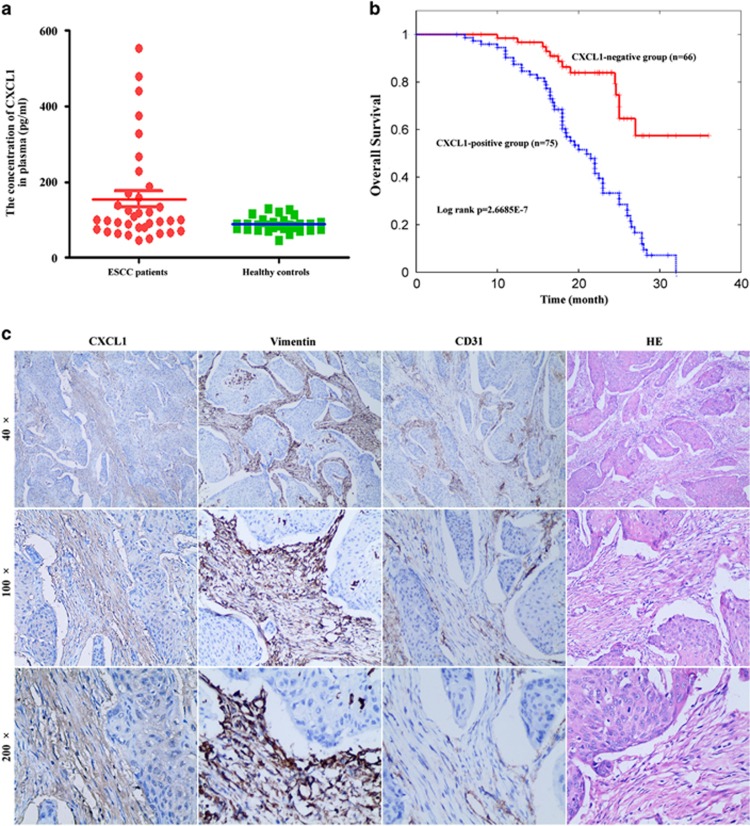
CXCL1 expressed in CAFs is an independent prognostic factor of ESCC patients treated with chemoradiotherapy. (**a**) The concentration of CXCL1 in plasma of ESCC patients (*n*=35) and in healthy controls (*n*=29) by ELISA. *P*=0.04207. (**b**) Kaplan–Meier analysis showed overall survival of ESCC patients with positive CXCL1 expression in CAFs (*n*=75) was significantly poorer than those CXCL1-negative patients in CAFs (*n*=66) after chemoradiotherapy. The grading of CXCL1 expression was described in ‘Materials and Methods’. (**c**) One representative result of CXCL1 expression in CAFs and in tumor tissues in 141 ESCC patients by IHC analysis. CD31 and vimentin were used for staining blood vessels and fibroblasts, respectively

As CAF-secreted CXCL1 was discovered as an important radiosensitizing target *in vitro* and *in vivo*, our study further explored its clinical significance by analysis of patients’ plasma and tumor tissues. By ELISA, the concentration of CXCL1 was found to be significantly higher in plasma samples of ESCC patients (*n*=35) compared with that in healthy controls (*n*=29), suggesting CXCL1 may serve as a potential tumor biomarker of ESCC patients (*P*=0.04207; Figure 7a). Furthermore, the association of CXCL1 expressed in CAFs and clinicopathological parameters, as well as overall survival of ESCC patients treated with chemoradiotherapy was statistically analyzed. By immunohistochemical (IHC) analysis, CXCL1 was found to be positively expressed in CAFs in 53.19% of ESCC patients (Table 1). The expression of CXCL1 in CAFs was significantly associated with tumor size (Table 1). Survival analysis showed the prognosis of ESCC patients with positive CXCL1 expression in CAFs was significantly poorer than those with negative CXCL1 expression in CAFs (Figures 7b and c). Univariate survival analysis demonstrated CXCL1 expression in CAFs, tumor size and lymph node metastasis were significantly associated with overall survival of ESCC patients treated with chemoradiotherapy (Table 2). Multivariate survival analysis showed only the expression of CXCL1 in CAFs was significantly associated with overall survival of ESCC patients treated with chemoradiotherapy, suggesting CXCL1 expressed in CAFs can be used as an independent prognostic factor of ESCC patients treated with chemoradiotherapy (Table 2).

## Discussion

Tumor radioresistance is heterogeneous and complicated.^22^ Previous studies from our lab and others have clarified multiple mechanisms underlying tumor radioresistance in ESCC.^5, 6, 7, 8, 9, 10^ However, these studies paid attention only to tumor cells. Several lines of evidences have suggested TME was closely associated with tumor response to chemoradiotherapy.^23, 24^ Particularly, CAFs, one major component of TME, transfer energy and biomass to support tumor growth and confer cell resistance to death stimuli.^12^ CAFs were involved in tumor radioresistance in glioma, prostate cancer and breast cancer.^25, 26, 27^ In ESCC, CAFs can be used as an independent prognostic factor and were associated with increased microvessel density, increased tumor-associated macrophages and epithelial to mesenchymal transition.^28, 29, 30^ However, the correlation of CAFs and tumor radioresistance has never been reported in ESCC.

As illustrated above, human chemokines showed strong activity in tumor cells and in the cross talk of tumor cells and their host microenvironment. In our study, the expression profiles of CAF-secreted human chemokines were analyzed by use of human chemokine/cytokine array. We found human chemokine CXCL1 was significantly highly expressed in CAFs compared with that in matched NFs as well as in tumor cells. The expressions of CXCL1 and its receptor CXCR2 were significantly increased following radiation in tumor cells, which may create a constitutively activated CXCL1/CXCR2 signaling. These results suggested CXCL1 may be involved in tumor radioresponse in ESCC. In human cancers such as gastric, colorectal and pancreatic cancer, CXCL1 was demonstrated to mediate angiogenesis and promote tumor progression.^31, 32, 33^ Shintani *et al.*^34^ reported CXCL1 was highly expressed in different SCC cell lines and tumor specimens, and associated with microvessel density, leukocyte infiltration and lymph node metastasis. CXCL1 was also found to be essential to activate epidermal growth factor receptor signaling and to increase proliferation of human dysplastic oral keratinocytes.^35^ Our study discovered CAF-secreted CXCL1 conferred significant radioresistance *in vitro* and *in vivo*.

When cultured in CAF medium, DNA damage repair following radiation was enhanced in tumor cells. The expression of *γ*-H2AX, a marker of DNA double-strand breaks, was decreased while the expressions of cellular DNA damage repair proteins were increased in tumor cells that were cultured in CAF medium. Inhibition of CAF-enhanced DNA damage repair significantly improved the radiosensitivity of tumor cells. As DNA is the main target of ionizing radiation, DNA damage repair is considered as a critical process that determines cell death or survival following radiation.^36^ Inhibition of DNA damage repair improved the radiosensitivity of several human cancers.^37, 38, 39^ We further dissected how CAF-secreted CXCL1 enhanced DNA damage repair following radiation in tumor cells. ROS are mitochondrial oxidative metabolism products or generated in cells that are experiencing bacterial invasion or chemoradiotherapy treatment.^19^ Under normal conditions, the status of ROS is tightly regulated by a variety of molecules that were responsible for the redox (reduction/oxidation) balance. Aberrant ROS status correlated with several human diseases including cancer, diabetes and aging as ROS was able to activate several cellular signaling pathways involved in DNA damage repair, cell proliferation, differentiation, survival and metabolism, and antioxidant, anti-inflammation response.^19^ SODs are the chief scavengers of ROS in cells.^20^ Several studies discovered SODs as important targets to reverse ROS-induced radioresponse.^40^ Our study uncovered CAF-secreted CXCL1 enhanced radiation-induced ROS increase. Furthermore, CAF-secreted CXCL1 enhanced DNA damage repair in a SOD1–ROS-dependent manner. Treatment with SOD1 protein reversed CXCL1-conferred radioresistance. These results suggested CAFs had important effects on redox system by which tumor radioresistance was conferred.

Previous study demonstrated CXCL1 was able to activate Erk signaling in cancer cells.^41^ Our study confirmed CAF-secreted CXCL1 activated Mek/Erk signaling pathway. As mentioned above, CAF-secreted CXCL1 enhanced radiation-induced ROS increase. It has been reported that ROS was able to activate Mek/Erk pathway by receptor and nonreceptor protein tyrosine kinases (PTKs) such as EGF/PDGF/insulin/ receptor PTKs and c-Src/JAK2 nonreceptor PTKs.^42^ Activated Mek/Erk pathway was proven to promote cell survival following radiation via multiple molecular mechanisms.^43, 44, 45^ Upon activation by phosphorylation, Erk increases the expressions of anti-apoptotic proteins including Bcl-xl, Mcl-1 and c-FlIps while inhibits the expressions of pro-apoptotic proteins such as Bad, Bim and caspase 9, thereby leading to tumor radioresistance.^46, 47, 48, 49, 50^ Erk signaling was also able to activate cellular DNA damage repair after radiation. Erk signaling was essential for radiation-induced cell cycle arrest and the accomplishment of DNA damage repair by upregulating the expressions of DNA repair proteins including DNA-PKcs, ERCC1, XRCC1 and XPC.^43, 45, 51, 52^ Furthermore, DNA damage sensor ATM was involved in radiation-induced Erk activation; in turn, inhibition of Erk activation attenuates radiation-induced ATM phosphorylation and the recruitment of ATM to DNA damage foci, suggesting there is a positive feedback loop involved in Erk/ATM signaling following radiation.^53^ Herein, our study found inhibition of Erk activation by CAF-secreted CXCL1 could also reverse tumor radioresistance.

Chronic inflammation is a high-risk factor to transfer esophagitis or gastritis to esophageal or gastric cancer, respectively.^54^ Human chemokines are key regulators that attract leukocytes to local inflammatory sites. CXCR2 ligands including CXCL1, 2, 3, CXCL5 and CXCL8 chemoattract pro-tumoral neutrophils promoting esophageal tumorigenesis.^54^ In an esophageal cancer cell line WHCO1, inhibition of CXCL1 signaling by transfection with CXCL1 RNAi or a specific antagonist of its receptor CXCR2 (SB225002) resulted in significant reduction in cell proliferation.^41^ In ESCC, CXCL1/CXCR2 signaling also enhanced the transcription of early growth response-1, which controlled cell growth, proliferation, differentiation and angiogenesis, and activated NF-*κ*B signaling pathway, which was significantly associated with poor treatment outcome of ESCC patients after chemoradiotherapy.^55, 56^ Our study found the cross talk of CAFs and tumor cells induced CXCL1 expression in an autocrine/paracrine signaling loop, which further enhanced the pathological roles of CXCL1 in ESCC. Collectively, our study highlighted CAF-secreted CXCL1 as an attractive target to radiosensitize ESCC *in vitro* and *in vivo*. Furthermore, CAF-secreted CXCL1 can be used as an independent prognostic factor of ESCC patients treated with chemoradiotherapy. The concentration of CXCL1 in plasma of ESCC patients was significantly higher than that in plasma of healthy controls, suggesting CXCL1 may be a potential tumor biomarker in ESCC. Our study for the first time discovered molecular mechanisms of radioresistance from the viewpoint of TME, and these findings may provide important implications for exploiting novel strategies against tumor radioresistance in ESCC.

## Materials and methods

### Isolation and culture of CAFs and matched NFs

To isolate stromal fibroblasts, tumor tissues and matched noncancerous esophageal tissues were obtained from two pathologically diagnosed ESCC patients who had not been treated with preoperative chemoradiotherapy before esophagectomy. Upon resection, tissues specimens were cut into as small pieces as possible, rinsed with PBS solution and then digested with 1 mg/ml collagenase type II for 2 h at 37 °C in 5% CO_2_/95% air. After filtration and centrifugation, cell precipitation was collected and seeded into 25 cm^2^ culture flask. Thirty minutes later, the medium was replaced with fresh medium to remove non-adherent cells (mainly tumor cells) to obtain pure fibroblasts, because adherent time of fibroblasts (<30 min) is much shorter than that of tumor cells (usually more than 1 h). After two to three passages, a unique homogeneity of stromal fibroblasts were obtained and cultured for further study. The fibroblasts isolated from tumor tissues were defined as CAFs and the fibroblasts from matched noncancerous esophageal tissues as NFs. Two pairs of CAFs and their matched NFs (CAF-1, CAF-2 and matched NF-1 and NF-2) were successfully isolated and used in our study.

### Culture of ESSC cells

The human ESCC cells KYSE-150 and KYSE-30 were obtained from American Type Culture Collection and cultured in RPMI-1640 medium (Gibco, Life Technologies Inc., Grand Island, NY, USA) supplemented with 10% fetal bovine serum (Gibco, Life Technologies Inc.) at 37 °C in 5% CO_2_/95% air.

### Preparation of the CM

ESCC cells, CAFs and NFs were seeded into 75 cm^2^ culture flask. After 48 h culture, when cells were grown at ~80% confluence, the culture medium was collected and centrifuged at 3000 r.p.m. at 4 °C for 30 min. The supernatant was collected as CM and kept at −80 °C until use. The CM from CAFs and NFs used in our study were defined as CAF medium and NF medium, respectively. Normal medium referred to fresh RPMI-1640 medium with 10% fetal bovine serum.

### Antibodies, agents, animals and tumor specimens of ESCC patients

Antibodies against p-ATM (Ser1981, #5883), Ku80 (#2180), Rad50 (#3247), p-RAD50 (Ser635, #14223), p-Chk2 (Thr68, #2197), Erk1/2 (#9102), p-Erk1/2 (Thr202/Tyr204, #4370), p-Mek1/2 (Ser217/221, #9154), E-cadherin (#3195S), Vimentin (5741S), SOD1 (#2770) and GAPDH (#2118) were purchased from Cell Signaling Technology (Beverly, MA, USA). Human SOD1 protein (#PHG 9214) was purchased from Epigentek Group Inc. (Buckingham, UK). Antibodies against *γ*-H2AX (S139, #ab26350), DNA-PKcs (#ab32566), p-DNA-PKcs (S2056, #ab124918), FAP (#ab28244), *α*-SMA (#ab5694) and Ki-67 (#ab92742) were purchased from Abcam (Cambridge, UK). Antibody against FSP-1 (#07-2274) was purchased from Millipore (Billerica, MA, USA). The inhibitor of CXCL1 receptor CXCR2 SB225002 (#S7651), ATM kinase inhibitor Ku55933 (#S1092) and Mek1/2 kinase inhibitor U0126 (#S1102) were purchased from Selleck (Houston, TX, USA).

Six-week-old female BALB/c nude mice were purchased from Vital River (Beijing, China) and maintained under standard conditions in Experimental Animal Center in Zhejiang Chinese Medicine University (Zhejiang, China). All of animal protocols in our study were in accordance with the institutional animal welfare guidelines of Zhejiang Chinese Medicine University. Tumor biopsy specimens of ESCC patients used for IHC analysis and human plasma samples were collected with the informed content of patients obtained. All of human studies in our study were in accordance with the guidelines of the Committees for Ethical Review of Research at Hangzhou Cancer Hospital. As shown in Supplementary Tables S1 and S2, the following information on patients participating in this study was provided including age, gender, TNM stage and tumor size.

### CXCL1 downregulation by transfection with siRNA against CXCL1

KYSE-150, KYSE-30 or CAFs were plated into six-well plates at a density of 1 × 10^5^ cells per well and cultured to adherent growth. Then, the mixture of CXCL1 siRNA (sense 5′–3′: GAUUAACUCUACCUGCACATT; antisense 5′–3′: UGUGCAGGUAGAGUUAAUCTT) and Lipofectamine 2000 (Hanbio Biotechnology Co., Ltd, Shanghai, China) was added into the cells. Six hours later, the medium was replaced with normal medium or CAF medium, and incubated for 24 h. The RNA was extracted to examine CXCL1 downregulation in cells that were transfected with CXCL1 siRNA. The cells that were transected with scrambled siRNA were used as a negative control.

### qRT-PCR analysis

Total RNA was extracted from tumor cells or CAFs using Trizol Reagent (Life Technologies, Carlsbad, CA, USA) following the manufacturer’s instructions. Reverse transcription was performed with Fermentas K1622 following the manufacturer’s instructions. qRT-PCR was conducted using SYBR green (Abgene, Epsom, UK) according to the manufacturer’s instructions. The PCR primers used in our study were synthesized by Invitrogen (San Diego, CA, USA) and shown in Supplementary Table S3.

### Western blotting analysis

Protein expression was examined by western blotting analysis. Briefly, cells receiving indicated treatments were collected by trypsin–EDTA exposure and washed twice with ice-cold PBS before adding into protein extraction buffer. Equal amount of proteins was fractionated on 12% SDS-PAGE gel and transferred to polyvinylidence difluoride membranes. The membranes were incubated with the indicated primary and secondary antibodies. Proteins were ultimately visualized by enhanced chemiluminescence and autoradiography (Thermo Scientific, Waltham, MA, UK).

### Determination of intracellular ROS level

Intracellular ROS level was determined with ROS assay kit (Beyotime, Shanghai, China) according to the manufacturer’s instructions. Briefly, cells were equipped with 10 mM DCFH-DA 30 min before radiation. Upon entry into cells, DCFH-DA is hydrolyzed by cellular esterase into DCFH, which then transfers to fluorescent DCF in response to radiation-induced ROS. Immunofluorescence images were taken using a confocal laser scanning microscope (CLSM, Nikon-A1 system, Dongjing, Japan).

### Clonogenic survival assay

Exponentially growing tumor cells were seeded into six-well plate under different conditions. After 24 h incubation, adhesive cells were exposed to radiation at 0, 2, 4, 6 and 8 Gy with an average dose rate of 100 cGy/min. Then, the cells were cultured for another 10 days at 37 °C in a 5% CO_2_ environment to allow colony formation. Only colonies containing ⩾50 cells were counted as clonogenic survivors. Unirradiated cells were chosen as a control.

### Enzyme-linked Immunosorbent assay

KYSE-150, KYSE-30, CAFs and NFs were seeded into 25 cm^2^ culture flask and cultured to adherent growth. Then, the medium was replaced with 4 ml fresh serum-free medium. After 48 h incubation, the culture medium was collected and centrifuged at 3000 r.p.m. at 4 °C for 30 min to obtain the supernatant. The detection of CXCL1 concentration in the supernatant of 10^6^ KYSE-150, KYSE-30, CAFs and NFs was performed with Human CXCL1 Quantikine ELISA Kit (R&D Systems, Minneapolis, MN, USA) according to the manufacturer’s instructions.

### Immunofluorescence analysis of *γ*-H2AX expression

Cells were seeded into six-well plate and cultured to adherent growth. After exposed to 8 Gy of radiaiton, cells were fixed with acetone/methanol (1:1), and permeabilized with 0.1% Triton X-100 in PBS. Nonspecific binding was blocked with 3% (m/v) bovine serum albumin (BSA) in PBS. Then, the cells were incubated with antibody against *γ*-H2AX for 2 h in PBS containing 0.1% (m/v) BSA. Indirect immunofluorescence was performed by incubation with Alexa Fluor 488-conjugated secondary antibodies (Invitrogen, Carlsbad, CA, USA). Immunofluorescence images were taken using a CLSM (Nikon-A1 system).

### Flow cytometry analysis of *γ*-H2AX expression

Cells were seeded into six-well plate and cultured to adherent growth. After exposed to 8 Gy of radiaiton, cells were collected, washed twice with ice-cold PBS and then fixed with 70% ethanol diluted in PBS. After kept at −20 °C overnight, cell samples was washed with 0.5% BSA in PBS and then incubated with Alexa Fluor 488-conjugated *γ*-H2AX antibody (CST, #9719) for 1 h before analysis by FCM (BD, Franklin Lakes, NJ, USA).

### Human cytokine/chemokine expression analysis

CAFs and NFs were seeded into 25 cm^2^ culture flask and cultured to adherent growth. Then, cells were washed twice with PBS and cultured in 4 ml fresh serum-free medium. After 24 h, culture medium was collected and centrifuged at 3000 r.p.m. at 4 °C for 30 min to obtain the conditioned medium of CAFs and NFs. The expression profile of human cytokines/chemokines was analyzed with human cytokine/chemokine antibody array (RayBiotech, Norcross, GA, USA) according to the manufacture’s instructions. Briefly, the conditioned medium was incubated with human cytokine/chemokine antibody array for 1 h at room temperature and then at 4 °C overnight. Then, the human cytokine/chemokine antibody was incubated with biotinylated antibody cocktail before with HRP–Streptavidin. Cytokines/chemokines were ultimately visualized by enhanced chemiluminescence and autoradiography (Thermo Scientific).

### Xenograft transplantation and therapy

To develop xenograft tumors, *in vitro*-growing KYSE-150, KYSE-30 and CAFs were collected by exposure to trypsin–EDTA and washed with ice-cold PBS. Then, KYSE-150 or KYSE-30 alone or combined with CAFs at a ratio of 8:1 (KYSE-150 or KYSE-30/CAFs) were implanted into the right flanks of female BALB/c nude mice. When xenograft tumors had reached a mean diameter of around 0.5 cm, mice were randomly assigned into different groups (six mice in each group). Tumors were treated with 12 Gy of radiation in six fractions, tumor injection of 1 *μ*g/ml CXCL1 antibody for 11 consecutive days alone or in their combinations (fractionated radiation was performed at day 1, 3, 5, 7, 9 and 11, the day when tumor injection of CXCL1 antibody began was defined as day 1). Tumors treated with only PBS was used as a control. Each animal was earmarked and followed individually throughout the experiments. Tumor volume (mm^3^) was calculated using the following formula: *V*(mm^3^)=*A*(mm) × *B*(mm)^2^/2, where *A* and *B* were the longest and widest diameter of tumor, respectively, and measured every 3 days by a caliper. At the end of the experiment, all mice were killed according to the institutional guidelines. Tumors were resected and weighed. IRs of tumor growth were calculated using the following equation: IR=100% × (mean tumor weight of control group − mean tumor weight of experimental group)/mean tumor weight of control group.

### IHC staining

IHC staining of CXCL1 was performed on paraffin-embedded sections of tumor biopsy specimens of ESCC patients. Briefly, sections of 4 *μ*m thick were deparaffinated and rehydrated trough a series of graded alcohols. Endogenous peroxidase activity was quenched with 3% (v/v) H_2_O_2_ for 20 min. Nonspecific binding was avoided by immersing sections into 3% BSA in PBS for 30 min at room temperature. Then, the sections were incubated with anti-CXCL1 primary antibody and HPR-conjugated secondary antibody. The intensity of CXCL1 expression was graded as 0, negative; 1+, weak cytoplasmic staining; 2+, strong staining in <30% of CAFs cells; and 3+, strong staining in more than 30% of CAFs cells. 0 and 1+ were defined as CXCL1-negative; and 2+ and 3+ as CXCL1-positive. The slides were scored by a pathologist and two experienced researchers independently.

### Statistics analysis

All of the experiments in our study were independently performed in triplicate, and the data were presented as means±S.D. Statistical analyses were performed with SPSS software 16.0 (SPSS, Chicago, IL, USA). Survival curves were estimated by Kaplan–Meier method, and the difference between the curves were evaluated by log-rank tests. The Cox proportional hazards regression model was used for the univariate and multivariate survival analysis with 95% CI and *P*-value calculated. The other statistical analyses were performed with Student’s *t*-test. Differences among groups were considered statistically significant at a level of *P*<0.05.

## Figures and Tables

**Table 1:** The association of CXCL1 expression in CAFs and clinicopathological characteristics of ESCC patients

	**CXCL1(−)**	**CXCL1(+)**	***P***
*Sex*			
Male	39	36	0.188
Female	27	39	

*Age (years)*			
⩽63	19	26	0.455
>63	47	49	

*Depth of invasion*			
Mucosa	13	18	0.327
Submucosa	21	14	
Muscularis	20	29	
Adventitia	12	14	

*Lymph node metastasis*			
None	29	21	0.221
Present	37	54	

*Tumor size (cm)*			
⩽6.5	52	22	0.000
>6.5	14	53	

**Table 2:** The association of clinicopathological characteristics with overall survival of ESCC patients treated with chemoradiotherapy

**Parameters**	**Univariate analysis**		**Multivariate analysis**	
	**Hazard ratio (95% CI)**	***P*-value**	**Hazard ratio (95% CI)**	***P*-value**
Age (years)	1.005±(0.977–1.035)	0.718	1.004±(0.975–1.033)	0.798
Gender	1.596±(0.956–2.664)	0.074	1.712±(0.998–2.937)	0.061
Depth of invasion	0.92±(0.727–1.166)	0.492	0.894±(0.702–1.138)	0.362
Lymph node metastasis	1.339±(1.076–1.665)	0.009	1.089±(0.82–1.446)	0.557
Tumor size (cm)	1.191±(1.056–1.344)	0.005	1.094±(0.947–1.264)	0.221
CXCL1 expression in CAFs	4.269±(2.322–7.847)	0.000	3.347±(1.639–6.835)	0.001
