# Supplementary material for: CAF-secreted CXCL1 conferred radioresistance by regulating DNA damage response in a ROS-dependent manner in esophageal squamous cell carcinoma
Source: Cell Death Dis. 2017 May 18;8(5):e2790–. doi: 10.1038/cddis.2017.180 (PMC5520705; doi:10.1038/cddis.2017.180)
Supplement: Supplementary Information [file cddis2017180x1.doc]

**Supporting Information**

**Supplementary Results**

**Characterization of CAFs isolated from tumor tissues of ESCC patients**

By primary culture, two pairs of CAFs and NFs with elongated spindle shape were isolated from tumor tissues of ESCC patients and matched normal esophageal epithelial tissues, respectively. We found CAFs and NFs both highly expressed mesenchymal marker vimentin, but not expressed epithelial marker E-cadherin (Supplementary Fig. S1). Furthermore, CAFs-related markers including myofibroblast marker α-SMA, fibroblast-specific protein-1 (FSP-1) and fibroblast activator protein (FAP) were highly expressed in CAF-1 and CAF-2 compared with in their matched NF-1 and NF-2 (Supplementary Fig. S1). These results suggested CAF-1 and CAF-2 had been successfully isolated with the characteristics of CAFs retained and can be used to study their effect on tumor radioresponse.

**Supplementary Figure legends**

**Supplementary Figure. S1** **Characterization of CAFs isolated from tumor tissues of ESCC patients.** Western blotting analysis of the expressions of epithelial marker E-cadherin and CAFs-related markers including myofibroblast marker α-SMA, fibroblast-specific protein-1 (FSP-1), fibroblast activator protein (FAP) and mesenchymal marker vimentin, and the expression of Ki-67, a proliferation-associated protein only present in proliferating cells in CAFs and in matched NFs. GAPDH was used as loading control.

**Supplementary Figure. S2 Human cytokine/chemokine array**

**Supplementary Figure. S3 CAFs attenuated radiation-induced DNA damage.** A. and B. Immunofluorescence analysis and FCM analysis of γ-H2AX expression at different time points after 8 Gy of radiation in KYSE-150 and in KYSE-30 that were cultured in normal medium or CAFs medium for 24 h. *Magnification*: 40 ×.

**Supplementary Figure. S4 CAFs enhanced radiation-induced ROS increase.** The ROS level following radiation in KYSE-150 and in KYSE-30 that were cultured in normal medium or CAFs medium for 24 h by immunofluorescence analysis. *Magnification*: 10 ×.

**Supplementary Figure. S5 SOD1 was involved in CAFs-enhanced ROS increase following radiation.** The ROS level following radiation in KYSE-150 and in KYSE-30 that were cultured in normal medium or CAFs medium with or without 100 ng/ml human SOD1 protein for 24 h by immunofluorescence analysis. *Magnification*: 10 ×.

**Supplementary Figure. S6 Blockage of CAFs-secreted CXCL1 enhanced radiation-induced tumor growth inhibitory effect in xenograft tumor models.** The IR (inhibition rate) of treatment with tumor injection of 1 μg/ml CXCL1 antibody, fractionated radiation at a total dose of 12 Gy alone or their combinations in xenograft tumors.

**Supplementary Table S1 Basic characteristics of 141 primary ESCC patients.**

**Supplementary Table S2 Basic characteristics of 35 primary ESCC patients.**

**Supplementary** **Table S3. Gene primers for qRT-PCR analysis**

**Supplementary Table S1. Basic characteristics of 141 primary ESCC patients**

| Total number | 141 |
| --- | --- |
| Age (years) |  |
| median | 63 |
| range | 35-79 |
| Gender |  |
| Female | 45 |
| Male | 96 |
| T stage |  |
| 1 | 31 |
| 2 | 35 |
| 3 | 49 |
| 4 | 26 |
| N stage |  |
| 0 | 50 |
| 1 | 31 |
| 2 | 32 |
| 3 | 28 |
| Tumor size (cm) |  |
| median | 6.5 |
| range | 2.5-15 |

**Supplementary Table S2. Basic characteristics of 35 primary ESCC patients**

| Total number | 35 |
| --- | --- |
| Age (years) |  |
| median | 68 |
| range | 58-72 |
| Gender |  |
| Female | 21 |
| Male | 14 |
| T stage |  |
| 1 | 5 |
| 2 | 6 |
| 3 | 15 |
| 4 | 9 |
| N stage |  |
| 0 | 6 |
| 1 | 7 |
| 2 | 15 |
| 3 | 7 |
| Tumor size (cm) |  |
| median | 6.2 |
| range | 3.2-14 |

**Supplementary** **Table S3. Gene primers for qRT-PCR analysis**

| genes | Forward primer (5’-3’) | Reverse primer (5’-3’) |
| --- | --- | --- |
| CXCL1 | CTTGCCTCAATCCTGCATC | CCTTCTGGTCAGTTGGATTTG |
| CXCR2 | CCTTCCTGGGTACAGTGCTATT | ACCGATGTCTTCACTGTGCC |
| SOD1 | GGTGGGCCAAAGGATGAAGAG | CCACAAGCCAAACGACTTCC |
| GAPDH | ATGACCCCTTCATTGACCTCA | GAGATGATGACCCTTTTGGCT |
