# Supplementary figures and images for: CAF-secreted CXCL1 conferred radioresistance by regulating DNA damage response in a ROS-dependent manner in esophageal squamous cell carcinoma
Source: Cell Death Dis. 2017 May 18;8(5):e2790–. doi: 10.1038/cddis.2017.180 (PMC5520705; doi:10.1038/cddis.2017.180)

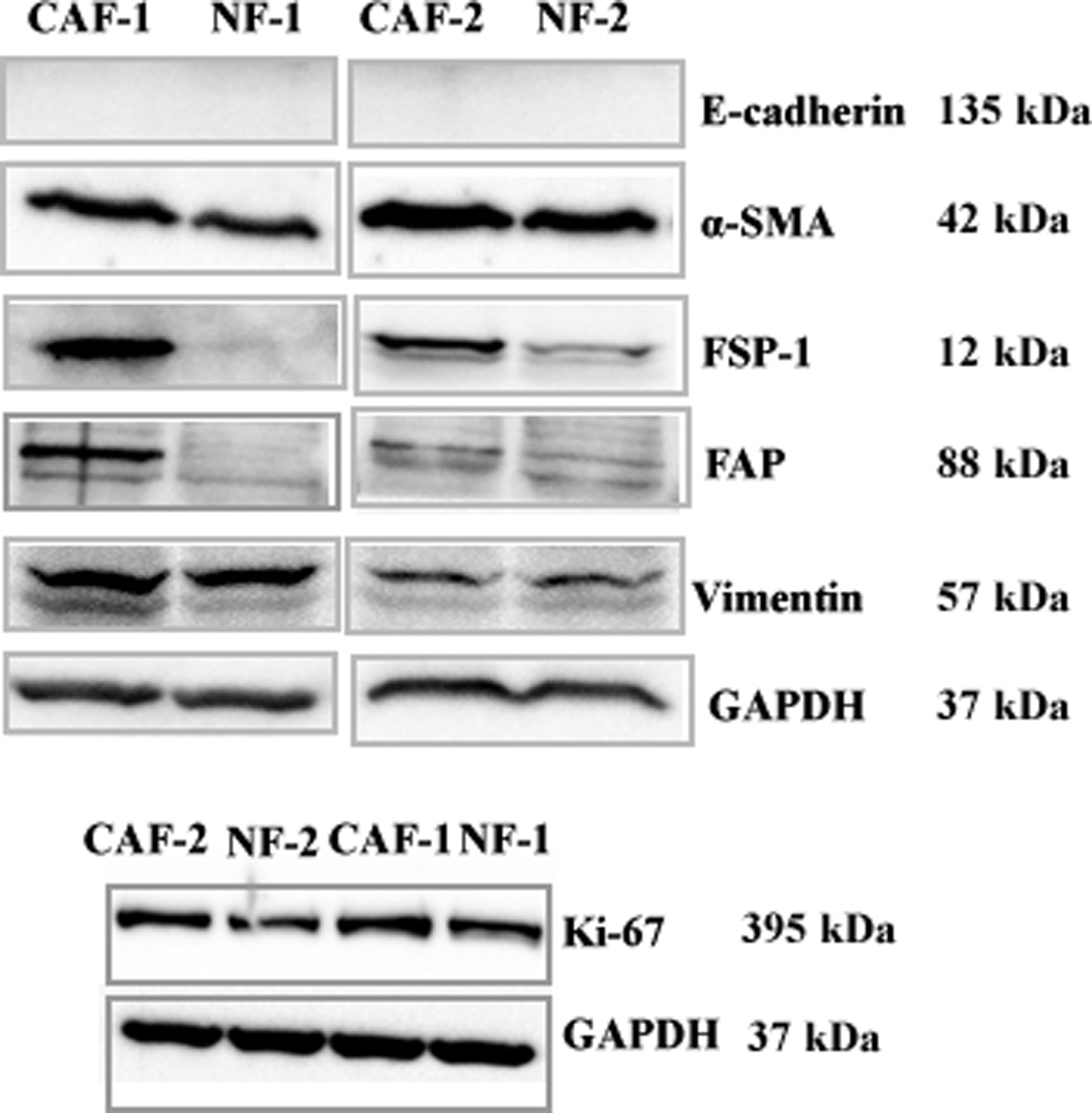

Supplement: Supplementary Figure 1 [file cddis2017180x2.tif]

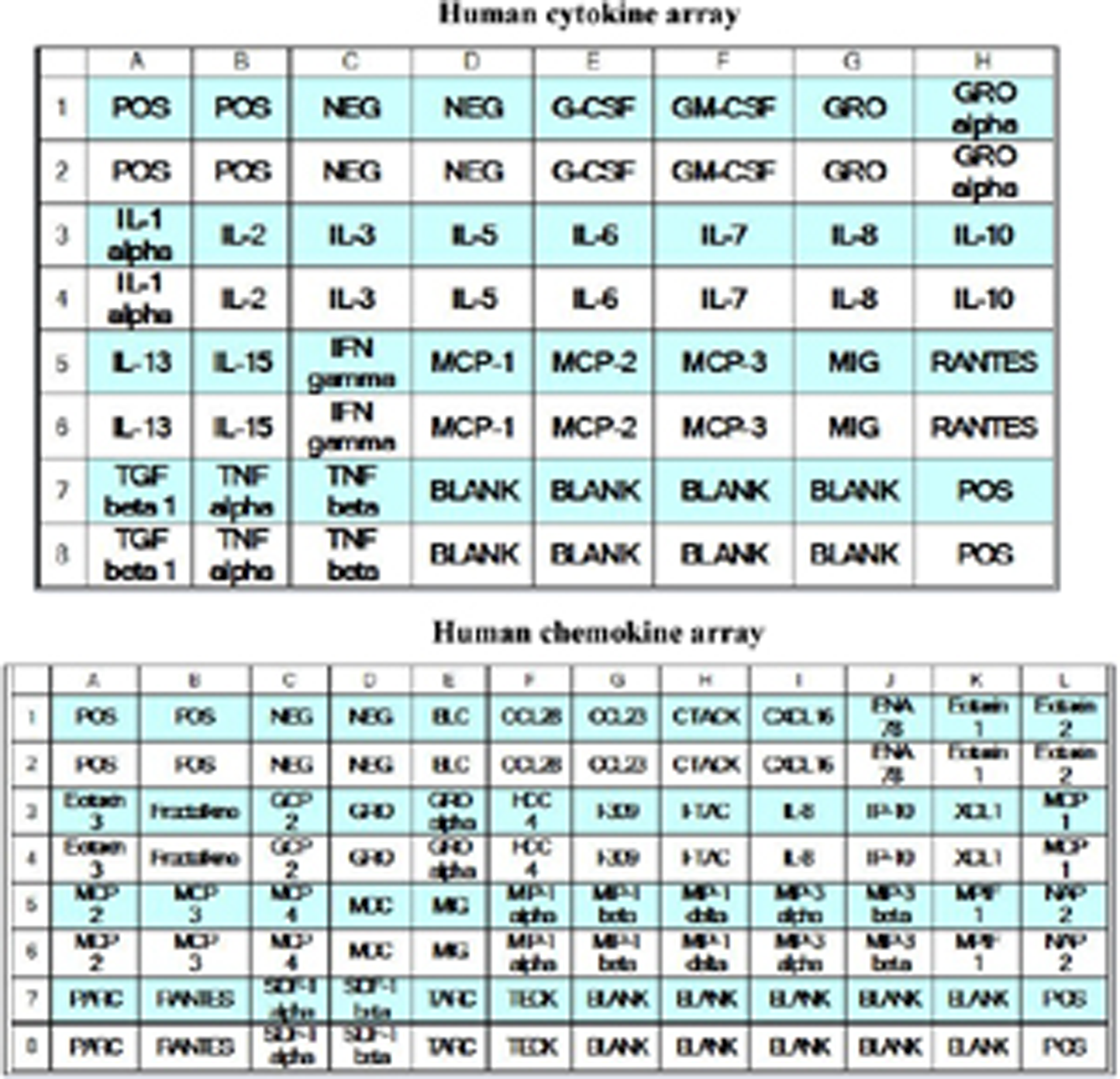

Supplement: Supplementary Figure 2 [file cddis2017180x3.tif]

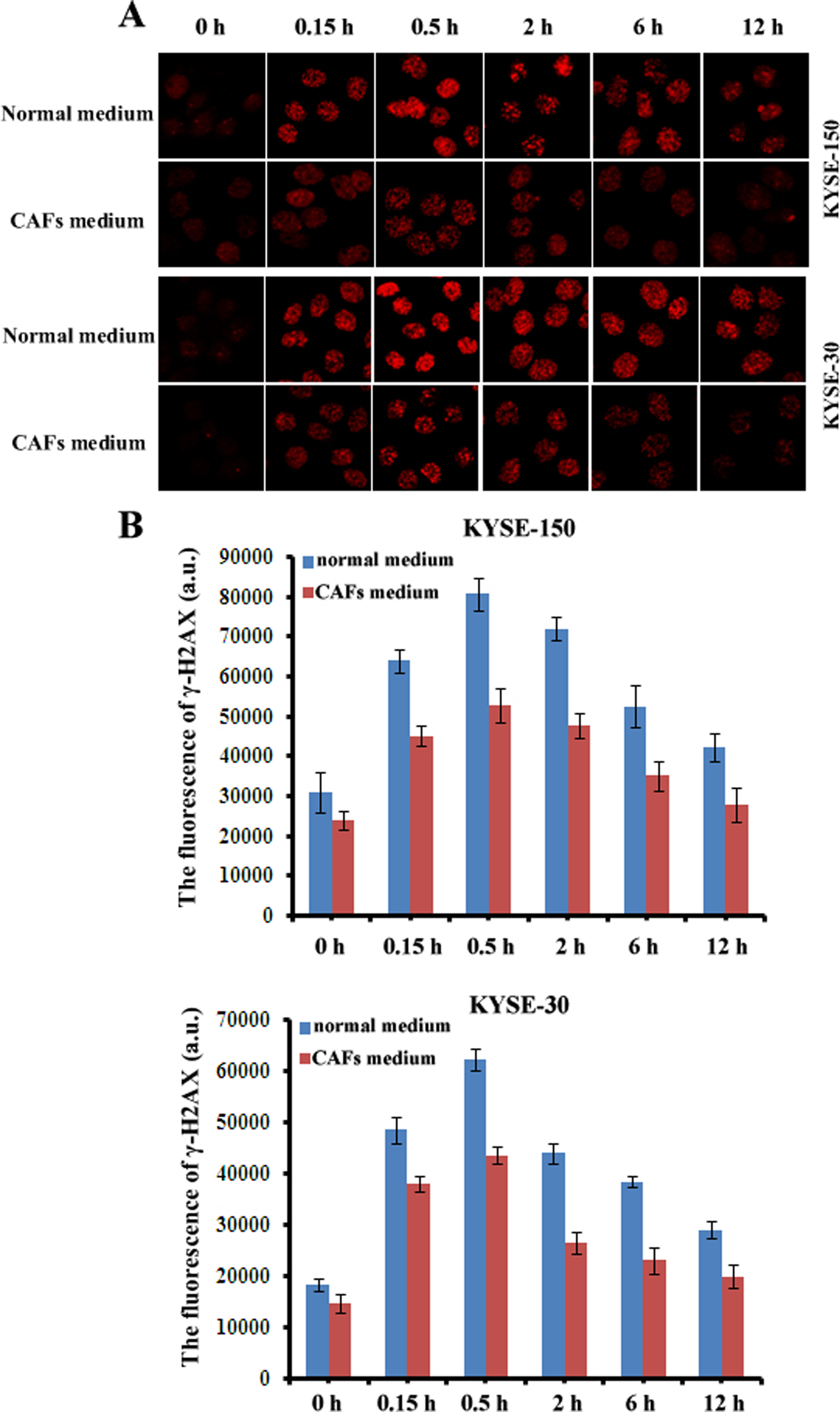

Supplement: Supplementary Figure 3 [file cddis2017180x4.tif]

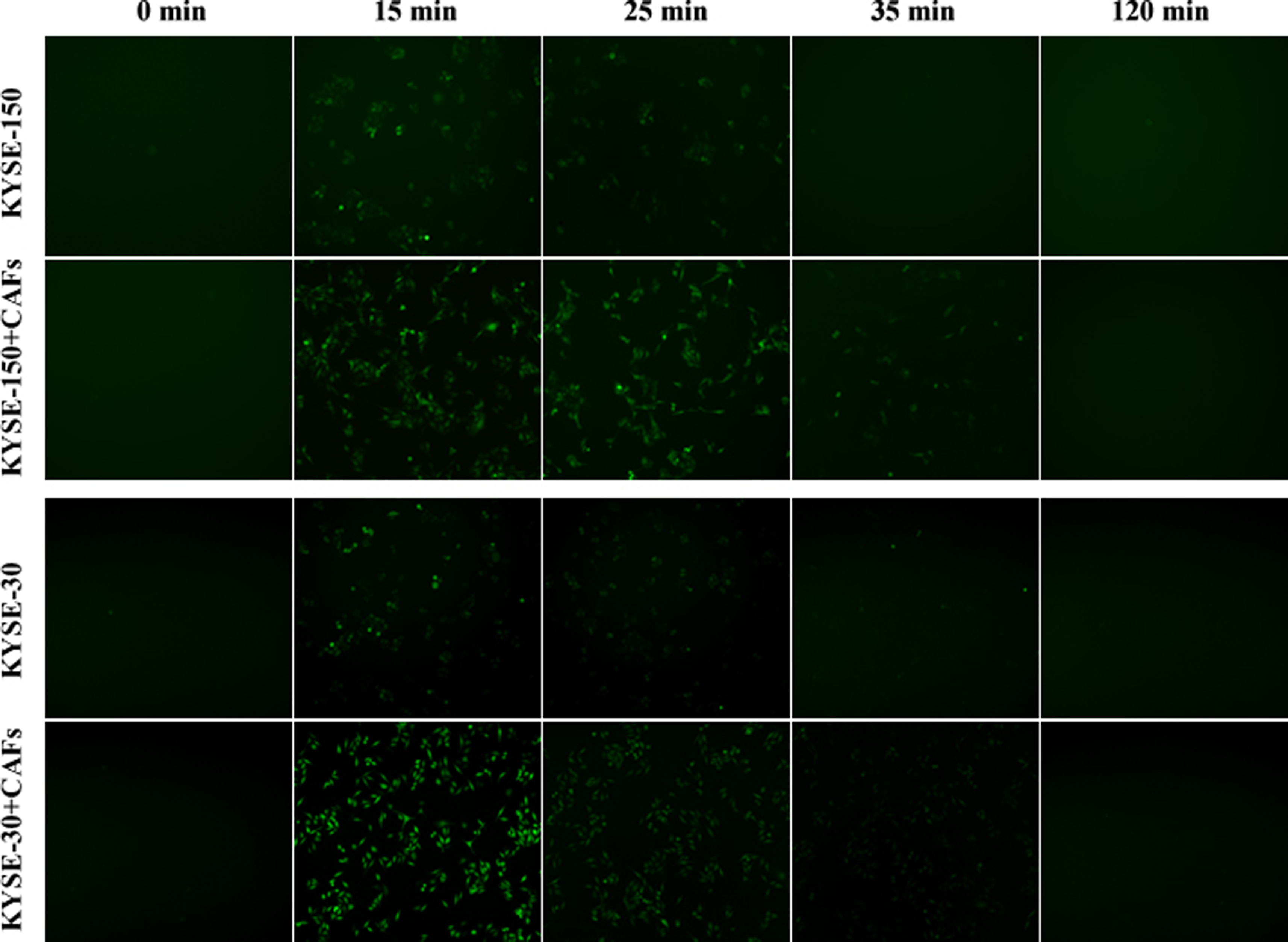

Supplement: Supplementary Figure 4 [file cddis2017180x5.tif]

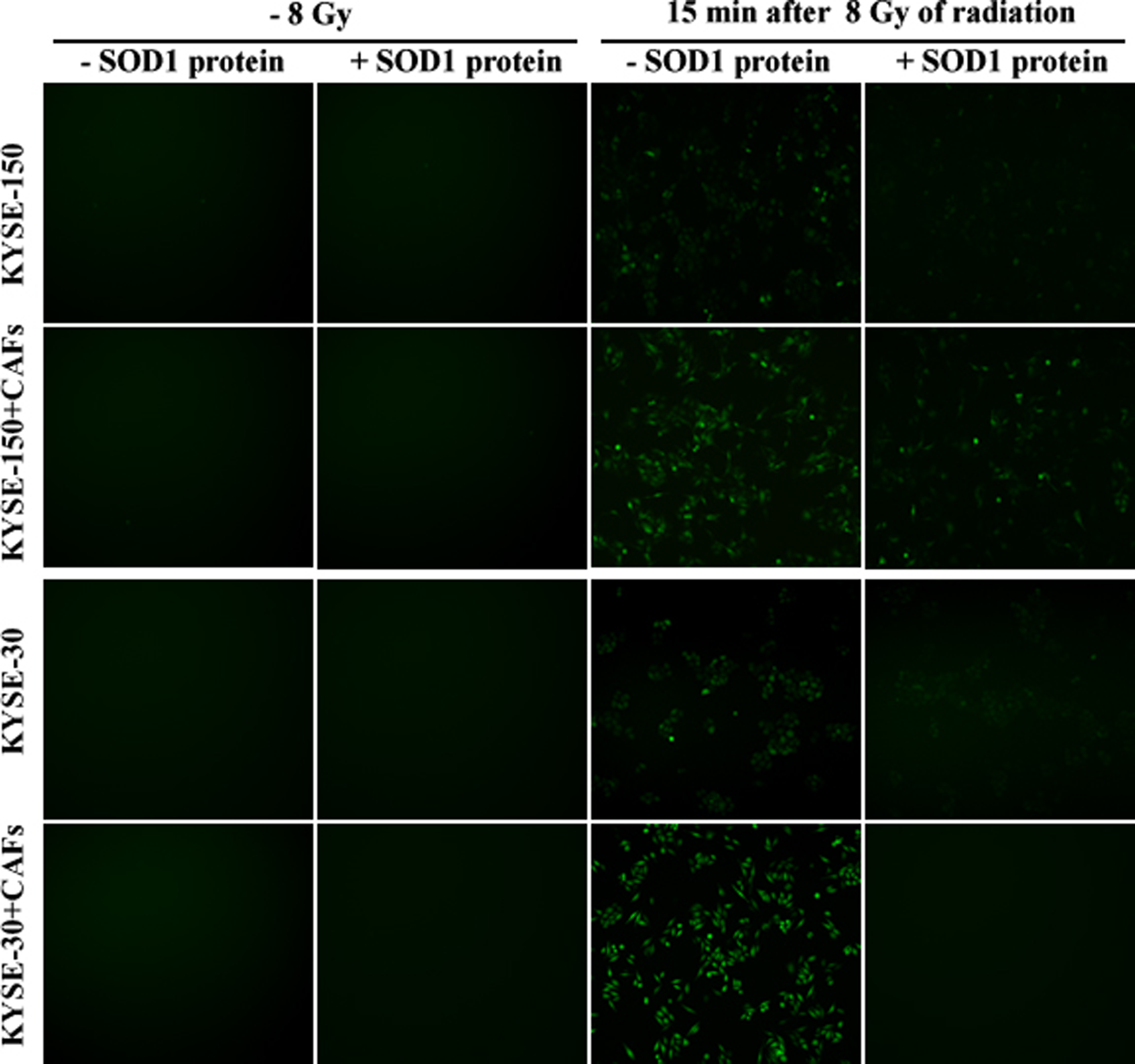

Supplement: Supplementary Figure 5 [file cddis2017180x6.tif]

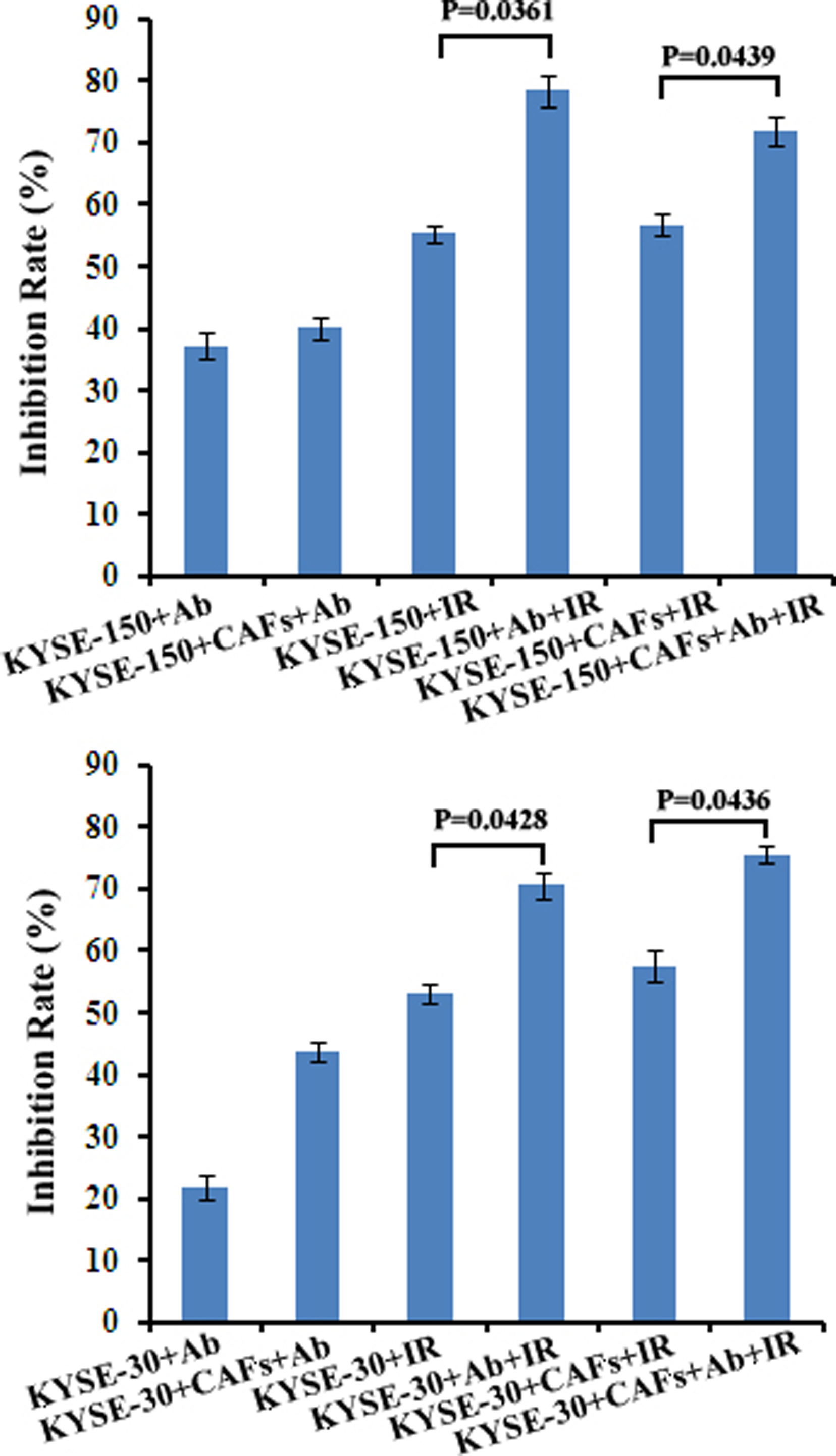

Supplement: Supplementary Figure 6 [file cddis2017180x7.tif]
